# A301 RELATIONSHIP BETWEEN INTRAHEPATIC IMMUNE CELL PROFILE AND MICROBIOME ACCORDING TO DISEASE SEVERITY IN OBESE PATIENTS WITH NON-ALCOHOLIC FATTY LIVER DISEASE

**DOI:** 10.1093/jcag/gwad061.301

**Published:** 2024-02-14

**Authors:** K J Schwenger, Y Ghorbani, L Chen, S Fischer, T Jackson

**Affiliations:** Medicine, University Health Network, Toronto, ON, Canada; University Health Network, Toronto, ON, Canada; Medicine, University Health Network, Toronto, ON, Canada; Medicine, University Health Network, Toronto, ON, Canada; Medicine, University Health Network, Toronto, ON, Canada

## Abstract

**Background:**

Non-alcoholic fatty liver disease (NAFLD) includes histology ranging from simple steatosis (SS) to non-alcoholic steatohepatitis (NASH), fibrosis and cirrhosis. The pathogenesis of NAFLD is multifactorial and includes several immune cell-mediated inflammatory processes. Recent research suggests a potential relationship between NAFLD, disease severity and the intestinal microbiome (IM). In particular, our previous work in NAFLD showed that lower abundance of fecal *Faecalibacterium prausnitzii* was associated with liver histology and gene expression. In addition, we found that Kupffer cell markers, hematopoietic cell marker cluster of differentiation and B cells were associated with liver histology.

**Aims:**

Knowing that *F prausnitzii* may have anti-inflammatory effect, we seek to determine if there was any association between fecal *F prausnitzii* and hepatic immune cells.

**Methods:**

Biochemical and anthropometric measurements as well fecal samples for IM analysis were collected in those with obesity undergoing bariatric surgery. Fecal shotgun metagenome sequencing to profile the intestinal microbiome Liver histology was assessed using Brunt scoring system. The frequency, location and phenotype of 8 immune cell subtypes were analyzed by multiplex immunofluorescence. Spearman correlation coefficients and partial Spearman correlation coefficients were used to evaluate the association between significant hepatic immune cells and fecal *F. prausnitzii*.

**Results:**

113 patients had both hepatic immune and IM data. Of the 113 patients, 30 had normal liver obese (NLO), 41 had SS and 42 had NASH. With regards to hepatic immune cells, those with NAFLD (SS and NASH) or NASH had significantly higher Helper T, CD4 and B cells and lower activated macrophages compared to NLO. In those with NAFLD or NASH, *F. prausnitzii* was significantly negatively correlated with Helper T cells (NAFLD, R= -0.25, p-value 0.009; NASH, R= -0.25, p-value 0.038). In those with NASH, *F. prausnitzii* was also significantly negatively correlated with CD4 cells (R= -0.31, p-value 0.009).

**Conclusions:**

Specific hepatic immune cells were associated with NAFLD and NASH with Helper T and CD4 cells being negatively correlated with *F. prausnitzii.* Knowing that these immune cells contribute to NAFLD pathogenesis, the relationship between fecal IM, hepatic immune cells and NAFLD require further exploration.

**Funding Agencies:**

CIHRCanadian Liver Foundation

